# 2,2,7,7,12,12,17,17-Octa­methyl-21,22,23,24-tetra­thia-2,7,12,17-tetra­germapenta­cyclo­[16.2.1.1^3,6^.1^8,11^.1^13,16^]tetra­cosa-3,5,8,10,13,15,18,20-octa­ene

**DOI:** 10.1107/S1600536812049720

**Published:** 2012-12-12

**Authors:** Guillaume Carel, Sonia Mallet-Ladeira, Ghassoub Rima, David Madec, Annie Castel

**Affiliations:** a Laboratoire Hétérochimie Fondamentale et Appliquée, UMR CNRS 5069, Université Paul Sabatier, 118 route de Narbonne, 31062 Toulouse Cedex 9, France; b Université de Toulouse, UPS, Institut de Chimie de Toulouse FR2599, 118 route de Narbonne, 31062 Toulouse Cedex 9, France

## Abstract

The title compound, [Ge_4_(CH_3_)_8_(C_4_H_2_S)_4_], crystallizes with one-half mol­ecule in the asymmetric unit, the whole mol­ecule being generated by inversion symmetry. The dihedral angle between adjacent thio­phene rings is 72.84 (14)°. In the crystal, mol­ecules are linked by C—H⋯π inter­actions, leading to the formation of chains along [100].

## Related literature

For a review concerning aryl- and heteroaryl­germanes, see: Spivey & Diaper (2003 ▶). For syntheses and structures of heteroaryl­germanes, see: Hockemeyer, Castel *et al.* (1997 ▶); Barrau *et al.* (1997 ▶); König & Rödel (1997 ▶). For properties of heteroaryl­germanes, see: Hockemeyer, Valentin *et al.* (1997 ▶). For a description of the Cambridge Structural Database, see: Allen (2002 ▶).
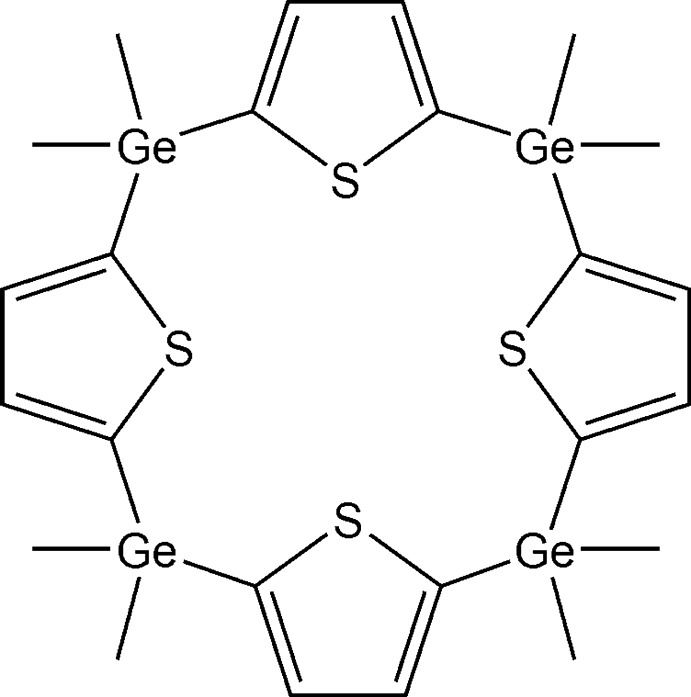


## Experimental

### 

#### Crystal data

[Ge_4_(CH_3_)_8_(C_4_H_2_S)_4_]
*M* *_r_* = 739.22
Monoclinic, 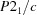

*a* = 6.6211 (4) Å
*b* = 12.6668 (7) Å
*c* = 18.3413 (11) Å
β = 90.698 (4)°
*V* = 1538.14 (16) Å^3^
*Z* = 2
Mo *K*α radiation
μ = 4.15 mm^−1^
*T* = 193 K
0.20 × 0.06 × 0.02 mm

#### Data collection

Bruker APEXII diffractometer
Absorption correction: multi-scan (*SADABS*; Bruker, 2006 ▶) *T*_min_ = 0.741, *T*_max_ = 0.922
34763 measured reflections
4222 independent reflections
3102 reflections with *I* > 2σ(*I*)
*R*_int_ = 0.065

#### Refinement

*R*[*F* ^2^ > 2σ(*F* ^2^)] = 0.030
*wR*(*F* ^2^) = 0.064
*S* = 1.01
4222 reflections
149 parameters
H-atom parameters constrained
Δρ_max_ = 0.44 e Å^−3^
Δρ_min_ = −0.37 e Å^−3^

### 

Data collection: *APEX2* (Bruker, 2006 ▶); cell refinement: *APEX2* and *SAINT* (Bruker, 2006 ▶); data reduction: *SAINT*; program(s) used to solve structure: *SHELXS97* (Sheldrick, 2008 ▶); program(s) used to refine structure: *SHELXL97* (Sheldrick, 2008 ▶); molecular graphics: *SHELXTL* (Sheldrick, 2008 ▶); software used to prepare material for publication: *SHELXTL* and *publCIF* (Westrip, 2010 ▶).

## Supplementary Material

Crystal structure: contains datablock(s) global, I. DOI: 10.1107/S1600536812049720/su2536sup1.cif

Structure factors: contains datablock(s) I. DOI: 10.1107/S1600536812049720/su2536Isup2.hkl

Additional supplementary materials:  crystallographic information; 3D view; checkCIF report

## Figures and Tables

**Table 1:** Hydrogen-bond geometry (Å, °) *Cg*1 is the centroid of the S1/C3–C6 ring.

*D*—H⋯*A*	*D*—H	H⋯*A*	*D*⋯*A*	*D*—H⋯*A*
C10—H10⋯*Cg*1^i^	0.95	2.82	3.606 (4)	141

Symmetry code: (i) 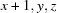.

**Fig. 1.**
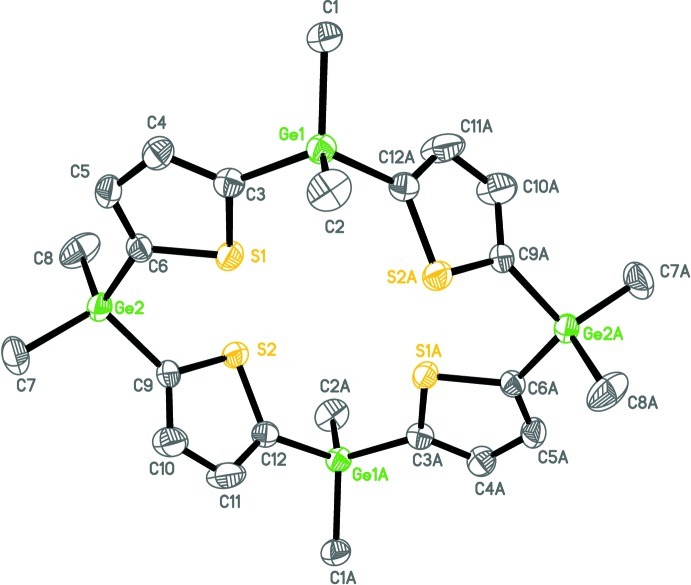
The molecular structure of the title molecule, with the atom numbering. The displacement ellipsoids are drawn at the 50% probability level. H atoms have been omitted for clarity. [Symmetry code: (A) -x + 1, -y + 1, -z + 1]

**Fig. 2.**
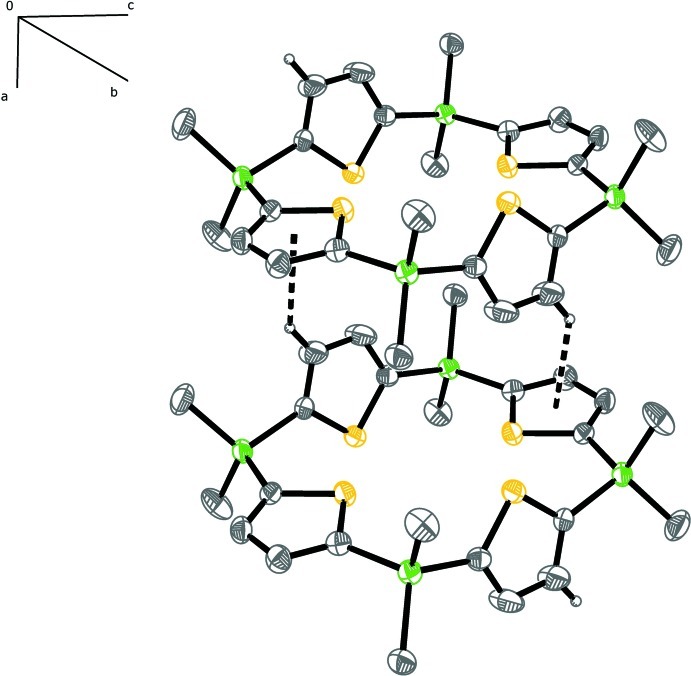
A partial view of the crystal packing of the title compound, showing the C—H··· π interactions (dashed lines; see Table 1 for details). H atoms not involved in these interactions have been omitted for clarity.

## supplementary crystallographic information

### Comment

Calix[4]thiophenes, sulfur-based analogues of calixarenes are of great importance for their uses in supramolecular chemistry. On the other hands, various hetero-calix[4]thiophenes in which group 14 atoms such as Si, Ge and Sn replace carbon atoms in the cyclic backbone have been prepared and characterized. However, to the best of our knowledge, no crystallographic data concerning germa-calixarene derivatives has far been reported so far (Cambridge Structural Database, V5.33, last update Aug. 2012; Allen, 2002).

The asymmetric unit of the title compound contains one half-molecule, the other half being related by a crystallographic inversion center (Fig. 1). In the asymmetric unit, the dihedral angle between adjacent thiophene rings is 72.84 (14)°. It is noteworthy that a C–H···π interaction between the hydrogen H10 and the π cloud of the thiophene ring S1/C3—C6 is observed giving stacks of the title compound along the *a* axis (Table 1 and Fig.2).

### Experimental

The title compound was prepared according to the following procedure:

In a first step, to a solution of thiophene (5.09 g, 60 mmol) and TMEDA (9.10 ml, 60 mmol) in dry diethyl ether (150 ml) was added a solution of *n*-BuLi (37.50 ml, 60 mmol, 1.6 *M* in hexanes). The mixture was stirred for 2 h at room temperature. A solution of Me_2_GeCl_2_ (5.20 g, 30 mmol) in dry diethyl ether (30 ml) was added slowly, the mixture was stirred for an additional 2 h. The reaction mixture was then filtered and the solvents removed by evaporation under reduced pressure. The residue was distillated to afford Me_2_Ge(C_4_H_3_S)_2_ (4.90 g, 61% yield).

In a second step, to a solution of Me_2_Ge(C_4_H_3_S)_2_ (2.69 g, 10 mmol) and TMEDA (3.0 ml, 20 mmol) in dry pentane (150 ml) cooled to 193 K was slowly added a solution of *n*-BuLi (12.50 ml, 20 mmol, 1.6 *M* in hexanes). The mixture was allowed to rise to room temperature and stirred for 2 h. To the formed precipitate in suspension was slowly added at 233 K a solution of Me_2_GeCl_2_ (1.75 g, 10 mmol) in dry pentane (50 ml). The mixture was allowed to rise to room temperature and stirred for 1h, and one additional hour at reflux. The reaction mixture was filtered and the solvents removed by evaporation under reduced pressure. The solid was washed by pentane. Crystals of the title compound were obtained by slow evaporation of a solution in CH_2_Cl_2_. Both the intermediate and the title compound were fully characterized, and spectroscopic and other data are available in the archived CIF.

### Refinement

All the H atoms were included in calculated positions and treated as riding atoms: C—H = 0.95 Å (aromatic), and 0.98 Å (methyl) with *U*_iso_(H) = 1.2*U*_eq_(aromatic) and *U*_iso_(H) = 1.5*U*_eq_(methyl).

### Crystal data

**Table:**

[Ge_4_(CH_3_)_8_(C_4_H_2_S)_4_]	*F*(000) = 736
*M**_r_* = 739.22	*D*_x_ = 1.596 Mg m^−^^3^
Monoclinic, *P*2_1_/*c*	Mo *K*α radiation, λ = 0.71073 Å
Hall symbol: -P 2ybc	Cell parameters from 5690 reflections
*a* = 6.6211 (4) Å	θ = 3.1–24.2°
*b* = 12.6668 (7) Å	µ = 4.15 mm^−^^1^
*c* = 18.3413 (11) Å	*T* = 193 K
β = 90.698 (4)°	Plate, colourless
*V* = 1538.14 (16) Å^3^	0.20 × 0.06 × 0.02 mm
*Z* = 2	

### Data collection

**Table:**

Bruker APEXII diffractometer	4222 independent reflections
Radiation source: fine-focus sealed tube	3102 reflections with *I* > 2σ(*I*)
Graphite monochromator	*R*_int_ = 0.065
phi and ω scans	θ_max_ = 29.4°, θ_min_ = 2.2°
Absorption correction: multi-scan (*SADABS*; Bruker, 2006)	*h* = −9→9
*T*_min_ = 0.741, *T*_max_ = 0.922	*k* = −17→17
34763 measured reflections	*l* = −25→25

### Refinement

**Table:**

Refinement on *F*^2^	Primary atom site location: structure-invariant direct methods
Least-squares matrix: full	Secondary atom site location: difference Fourier map
*R*[*F*^2^ > 2σ(*F*^2^)] = 0.030	Hydrogen site location: inferred from neighbouring sites
*wR*(*F*^2^) = 0.064	H-atom parameters constrained
*S* = 1.01	*w* = 1/[σ^2^(*F*_o_^2^) + (0.0251*P*)^2^ + 0.5305*P*] where *P* = (*F*_o_^2^ + 2*F*_c_^2^)/3
4222 reflections	(Δ/σ)_max_ = 0.001
149 parameters	Δρ_max_ = 0.44 e Å^−^^3^
0 restraints	Δρ_min_ = −0.37 e Å^−^^3^

### Special details

**Table:**

Experimental. Spectroscopic data for the intermediate: M.p.= 363 - 364 K/0.25 mm Hg. ^1^H NMR (300 MHz in CDCl_3_) δ, p.p.m.: 7.71–7.64 (m, 2H), 7.36–7.31 (m, 2H), 7.31–7.34 (m, 2H), 0.85 (s, 6H). ^13^C NMR (75 MHz in CDCl_3_) δ, p.p.m.: 138.1, 133.9, 130.4, 128.1, -0.1. MS (EI, 70 eV) m/*z*= 270 (*M*^+.^). UV: λ_max_= 235 nm, log ε= 1.41. IR (Nujol, cm^-1^): 3100, 3073, 2976, 2907, 1497, 1402, 1214, 1080, 974, 848, 831, 807, 746, 704. Anal. Found: C, 44.62; H, 4.57. Calc. for C_10_H_12_S_2_Ge: C, 44.68; H, 4.47.Spectroscopic data for the title compound: M.p.: 389 - 390 K(dec.). ^1^H NMR (300 MHz in CDCl_3_) δ, p.p.m.: 7.31 (s, 8H), 0.78 (s, 24H). ^13^C NMR (75 MHz in CDCl_3_) δ, p.p.m.: 143.6, 134.9, 1.2. MS (EI, 70 eV) m/*z*= 740 (*M*^+.^). UV: λ_max_= 247 nm, log ε = 4.6. IR (Nujol, cm^-1^): 2960, 2915, 1643, 1490, 1406, 1270, 1240, 1200, 985, 953, 836, 802, 738. Anal. Found: C, 38.85; H, 4.42. Calc. for C_24_H_32_S_4_Ge_4_: C, 39.00; H, 4.33.
Geometry. All e.s.d.'s (except the e.s.d. in the dihedral angle between two l.s. planes) are estimated using the full covariance matrix. The cell e.s.d.'s are taken into account individually in the estimation of e.s.d.'s in distances, angles and torsion angles; correlations between e.s.d.'s in cell parameters are only used when they are defined by crystal symmetry. An approximate (isotropic) treatment of cell e.s.d.'s is used for estimating e.s.d.'s involving l.s. planes.
Refinement. Refinement of *F*^2^ against ALL reflections. The weighted *R*-factor *wR* and goodness of fit *S* are based on *F*^2^, conventional *R*-factors *R* are based on *F*, with *F* set to zero for negative *F*^2^. The threshold expression of *F*^2^ > σ(*F*^2^) is used only for calculating *R*-factors(gt) *etc*. and is not relevant to the choice of reflections for refinement. *R*-factors based on *F*^2^ are statistically about twice as large as those based on *F*, and *R*- factors based on ALL data will be even larger.

### Fractional atomic coordinates and isotropic or equivalent isotropic displacement parameters (Å^2^)

**Table:**

	*x*	*y*	*z*	*U*_iso_*/*U*_eq_	
Ge1	0.09401 (4)	0.22725 (2)	0.492665 (15)	0.02782 (8)	
Ge2	0.54568 (4)	0.48911 (2)	0.256324 (15)	0.02951 (8)	
S1	0.37473 (10)	0.38100 (5)	0.39987 (3)	0.03005 (15)	
S2	0.62663 (10)	0.65368 (6)	0.38758 (4)	0.03257 (16)	
C1	−0.1957 (4)	0.2081 (2)	0.48350 (16)	0.0387 (7)	
H1A	−0.2595	0.2758	0.4714	0.058*	
H1B	−0.2490	0.1820	0.5297	0.058*	
H1C	−0.2251	0.1569	0.4448	0.058*	
C2	0.2337 (5)	0.0964 (2)	0.51710 (17)	0.0461 (8)	
H2A	0.2128	0.0450	0.4778	0.069*	
H2B	0.1800	0.0679	0.5626	0.069*	
H2C	0.3784	0.1103	0.5232	0.069*	
C3	0.1957 (4)	0.2830 (2)	0.40179 (14)	0.0295 (6)	
C4	0.1417 (4)	0.2561 (2)	0.33170 (15)	0.0366 (7)	
H4	0.0444	0.2033	0.3203	0.044*	
C5	0.2455 (4)	0.3148 (2)	0.27812 (15)	0.0346 (6)	
H5	0.2239	0.3049	0.2273	0.042*	
C6	0.3792 (4)	0.3868 (2)	0.30595 (13)	0.0281 (5)	
C7	0.7184 (5)	0.4178 (3)	0.18768 (17)	0.0544 (9)	
H7A	0.7990	0.4701	0.1615	0.082*	
H7B	0.6351	0.3779	0.1528	0.082*	
H7C	0.8086	0.3693	0.2140	0.082*	
C8	0.3709 (5)	0.5915 (2)	0.20864 (18)	0.0535 (9)	
H8A	0.2960	0.6308	0.2455	0.080*	
H8B	0.2756	0.5548	0.1762	0.080*	
H8C	0.4528	0.6405	0.1802	0.080*	
C9	0.7126 (4)	0.5572 (2)	0.32987 (13)	0.0274 (5)	
C10	0.9108 (4)	0.5395 (3)	0.34415 (18)	0.0493 (8)	
H10	0.9887	0.4889	0.3186	0.059*	
C11	0.9916 (4)	0.6038 (3)	0.40107 (18)	0.0525 (9)	
H11	1.1286	0.6003	0.4169	0.063*	
C12	0.8553 (4)	0.6707 (2)	0.43070 (14)	0.0291 (6)	

### Atomic displacement parameters (Å^2^)

**Table:**

	*U*^11^	*U*^22^	*U*^33^	*U*^12^	*U*^13^	*U*^23^
Ge1	0.02892 (14)	0.02620 (14)	0.02832 (15)	−0.00283 (11)	−0.00068 (11)	0.00038 (11)
Ge2	0.03170 (15)	0.03462 (16)	0.02218 (14)	−0.00246 (12)	−0.00042 (11)	0.00054 (12)
S1	0.0324 (3)	0.0329 (4)	0.0247 (3)	−0.0058 (3)	−0.0027 (3)	0.0015 (3)
S2	0.0283 (3)	0.0388 (4)	0.0305 (4)	0.0048 (3)	−0.0027 (3)	−0.0058 (3)
C1	0.0322 (14)	0.0451 (17)	0.0386 (16)	−0.0069 (13)	−0.0029 (12)	−0.0004 (13)
C2	0.0519 (18)	0.0328 (16)	0.053 (2)	0.0011 (14)	−0.0074 (15)	0.0035 (14)
C3	0.0300 (13)	0.0257 (13)	0.0330 (14)	−0.0012 (11)	0.0004 (11)	−0.0011 (11)
C4	0.0399 (15)	0.0337 (15)	0.0362 (16)	−0.0078 (12)	0.0001 (13)	−0.0090 (12)
C5	0.0393 (15)	0.0384 (15)	0.0261 (14)	−0.0031 (12)	0.0010 (12)	−0.0068 (12)
C6	0.0275 (12)	0.0310 (14)	0.0260 (13)	0.0034 (11)	0.0025 (10)	−0.0016 (11)
C7	0.0522 (19)	0.074 (2)	0.0372 (18)	−0.0103 (18)	0.0156 (15)	−0.0179 (17)
C8	0.059 (2)	0.0482 (19)	0.053 (2)	−0.0040 (16)	−0.0258 (17)	0.0174 (16)
C9	0.0295 (13)	0.0307 (13)	0.0221 (13)	−0.0006 (11)	0.0026 (10)	0.0021 (10)
C10	0.0350 (15)	0.0518 (19)	0.061 (2)	0.0096 (14)	−0.0035 (15)	−0.0294 (16)
C11	0.0293 (15)	0.058 (2)	0.070 (2)	0.0078 (14)	−0.0124 (15)	−0.0270 (18)
C12	0.0280 (12)	0.0285 (14)	0.0307 (14)	−0.0006 (11)	0.0004 (11)	0.0005 (11)

### Geometric parameters (Å, º)

**Table:**

Ge1—C12^i^	1.935 (3)	C3—C4	1.373 (4)
Ge1—C3	1.938 (3)	C4—C5	1.417 (4)
Ge1—C1	1.939 (3)	C4—H4	0.9500
Ge1—C2	1.948 (3)	C5—C6	1.366 (3)
Ge2—C7	1.935 (3)	C5—H5	0.9500
Ge2—C9	1.936 (2)	C7—H7A	0.9800
Ge2—C6	1.936 (3)	C7—H7B	0.9800
Ge2—C8	1.939 (3)	C7—H7C	0.9800
S1—C3	1.718 (3)	C8—H8A	0.9800
S1—C6	1.725 (3)	C8—H8B	0.9800
S2—C12	1.714 (2)	C8—H8C	0.9800
S2—C9	1.718 (3)	C9—C10	1.354 (4)
C1—H1A	0.9800	C10—C11	1.424 (4)
C1—H1B	0.9800	C10—H10	0.9500
C1—H1C	0.9800	C11—C12	1.356 (4)
C2—H2A	0.9800	C11—H11	0.9500
C2—H2B	0.9800	C12—Ge1^i^	1.935 (3)
C2—H2C	0.9800		
			
C12^i^—Ge1—C3	108.81 (11)	C5—C4—H4	123.3
C12^i^—Ge1—C1	108.00 (11)	C6—C5—C4	114.1 (2)
C3—Ge1—C1	108.94 (11)	C6—C5—H5	122.9
C12^i^—Ge1—C2	108.88 (12)	C4—C5—H5	122.9
C3—Ge1—C2	109.81 (12)	C5—C6—S1	109.0 (2)
C1—Ge1—C2	112.32 (13)	C5—C6—Ge2	129.9 (2)
C7—Ge2—C9	108.94 (12)	S1—C6—Ge2	120.97 (14)
C7—Ge2—C6	109.73 (13)	Ge2—C7—H7A	109.5
C9—Ge2—C6	107.11 (10)	Ge2—C7—H7B	109.5
C7—Ge2—C8	111.90 (15)	H7A—C7—H7B	109.5
C9—Ge2—C8	110.41 (12)	Ge2—C7—H7C	109.5
C6—Ge2—C8	108.64 (12)	H7A—C7—H7C	109.5
C3—S1—C6	94.08 (13)	H7B—C7—H7C	109.5
C12—S2—C9	94.39 (12)	Ge2—C8—H8A	109.5
Ge1—C1—H1A	109.5	Ge2—C8—H8B	109.5
Ge1—C1—H1B	109.5	H8A—C8—H8B	109.5
H1A—C1—H1B	109.5	Ge2—C8—H8C	109.5
Ge1—C1—H1C	109.5	H8A—C8—H8C	109.5
H1A—C1—H1C	109.5	H8B—C8—H8C	109.5
H1B—C1—H1C	109.5	C10—C9—S2	109.0 (2)
Ge1—C2—H2A	109.5	C10—C9—Ge2	127.2 (2)
Ge1—C2—H2B	109.5	S2—C9—Ge2	123.75 (14)
H2A—C2—H2B	109.5	C9—C10—C11	113.7 (3)
Ge1—C2—H2C	109.5	C9—C10—H10	123.2
H2A—C2—H2C	109.5	C11—C10—H10	123.2
H2B—C2—H2C	109.5	C12—C11—C10	114.0 (2)
C4—C3—S1	109.4 (2)	C12—C11—H11	123.0
C4—C3—Ge1	128.7 (2)	C10—C11—H11	123.0
S1—C3—Ge1	121.86 (14)	C11—C12—S2	108.9 (2)
C3—C4—C5	113.3 (2)	C11—C12—Ge1^i^	126.73 (19)
C3—C4—H4	123.3	S2—C12—Ge1^i^	124.31 (14)

Symmetry code: (i) −*x*+1, −*y*+1, −*z*+1.

### Hydrogen-bond geometry (Å, º)

Cg1 is the centroid of the S1/C3–C6 ring.

**Table:**

*D*—H···*A*	*D*—H	H···*A*	*D*···*A*	*D*—H···*A*
C10—H10···*Cg*1^ii^	0.95	2.82	3.606 (4)	141

Symmetry code: (ii) *x*+1, *y*, *z*.
